# Production and Evaluation of Virus-Like Particles Displaying Immunogenic Epitopes of Porcine Reproductive and Respiratory Syndrome Virus (PRRSV)

**DOI:** 10.3390/ijms16048382

**Published:** 2015-04-14

**Authors:** Ambika Mosale Venkatesh Murthy, Yanyan Ni, Xiangjin Meng, Chenming Zhang

**Affiliations:** 1 Department of Biological Systems Engineering, Virginia Tech, Blacksburg, VA 24061, USA; E-Mail: ambika3011@gmail.com; 2 Center for Molecular Medicine and Infectious Disease, Department of Biomedical Sciences & Pathobiology, College of Veterinary Medicine, Virginia Tech, Blacksburg, VA 24060, USA; E-Mails: nyy7@vt.edu (Y.N.); xjmeng@vt.edu (X.M.)

**Keywords:** porcine reproductive and respiratory syndrome virus, PRRSV, vaccine, VLP, inclusion bodies

## Abstract

Porcine reproductive and respiratory syndrome (PRRS) is the most significant infectious disease currently affecting the swine industry worldwide. Several inactivated and modified live vaccines (MLV) have been developed to curb PRRSV infections. However, the efficacy and safety of these vaccines are unsatisfactory, and hence, there is a strong demand for the development of new PRRS universal vaccines. Virus-like particle (VLP)-based vaccines are gaining increasing acceptance compared to subunit vaccines, as they present the antigens in a more veritable conformation and are readily recognized by the immune system. Hepatitis B virus core antigen (HBcAg) has been successfully used as a carrier for more than 100 viral sequences. In this study, hybrid HBcAg VLPs were generated by fusion of the conserved protective epitopes of PRRSV and expressed in *E. coli*. An optimized purification protocol was developed to obtain hybrid HBcAg VLP protein from the inclusion bodies. This hybrid HBcAg VLP protein self-assembled to 23-nm VLPs that were shown to block virus infection of susceptible cells when tested on MARC 145 cells. Together with the safety of non-infectious and non-replicable VLPs and the low cost of production through *E. coli* fermentation, this hybrid VLP could be a promising vaccine candidate for PRRS.

## 1. Introduction

Porcine reproductive and respiratory syndrome (PRRS) emerged as a disastrous disease in the late 1980s. It was first identified in 1987 in the U.S. and in 1990 in Europe [1]. The disease accounts for more than 600 million dollars annual loss for the U.S. swine industry alone. It causes severe reproductive problems, such as poor farrowing rates, premature farrowings and increased stillbirths in sows and respiratory distress, such as pneumonia in piglets and growing pigs [2].

The causative agent, PRRS virus (PRRSV), is a single-stranded enveloped RNA virus of order *Nidovirales* and family *Arterviridae.* PRRSV can be grouped into two genotypes, namely European (Type 1) and North American (Type 2) [3]. Among all proteins encoded by the virus genome, several structural proteins of PRRSV are known to induce neutralizing antibodies, but GP5-induced neutralization antibodies perform a vital role in protection against infection [4,5]. The neutralization epitope, located in the middle of the GP5 sequence and corresponding to the B-cell epitope (^37^SHLQLIYNL^46^), is conserved among North American PRRSV isolates [6]. Two more pentadecapeptides (^117^LAALICFVIRLAKNC^131^ and ^149^KGRLYRWRSPVIIEK^163^) spanning GP5 are identified as immunodominant T-cell epitopes [7]. These T-cell epitopes are relatively conserved with at most two amino acid variations and are known to elicit an interferon-gamma response from peripheral blood mononuclear cells [7].

A variety of inactivated and modified live vaccines (MLVs) have been developed to prevent PRRSV infections. Inactivated PRRSV vaccines are considered ineffective, as they fail to prevent clinical signs and viremia caused by virus challenge, even with homologous strains [8]. Although MLVs are more effective when compared to inactivated vaccines in the reduction of clinical signs, they have several disadvantages. First, they confer only partial protection against heterologous strains. Second, they are unstable and can revert to virulent viruses under farm conditions. Third, vaccinated pigs shed infective virus, which demands vaccination of all pigs in the pen at the same time [9]. Therefore, it is critical to develop new generation PRRSV vaccines that can confer protection against field PRRS viruses.

Virus-like particles (VLPs) are gaining increasing acceptance as potential vaccine candidates, as they overcome the shortcomings associated with inactivated vaccines and MLVs [10]. VLPs are an expedient candidate for vaccine design, as they mimic the 3D structure of native viruses and are devoid of viral genome. They are known to stimulate a B-cell-mediated response, a CD4 cell proliferating response and cytotoxic T lymphocyte (CTL) responses. VLPs cross-link the B-cell receptors and efficiently reach the MHC class I pathway [11]. They can even target dendritic cells (DC), which are essential to invoke both humoral and cell-mediated immunity [12]. VLPs formed by hepatitis B core antigen (HBcAg) can act as an efficient carrier platform to display antigens (such as epitopes) unrelated to the VLP [11]. Poorly immunogenic B-cell and T-cell epitopes have been converted into a highly immunogenic malaria vaccine candidate by linkage of these epitopes to HBcAg VLPs [13]. Furthermore, a universal influenza A vaccine by fusing a conserved viral 23-amino-acid M2 peptide (M2e) with HBcAg has successfully completed a phase I clinical trial [14].

Unlike the two recent reports that described the use of VLPs containing the entire GP5 [15] or GP5 and M [16] as vaccines against PRRSV, the construction of HBcAg VLPs fused with the conserved protective epitopes of PRRSV is reported here. The hypothesis is that by displaying the conserved immunogenic epitopes of PRRSV on HBcAg VLPs, the vaccine would elicit focused and strong immune response towards those epitopes. The hybrid HBcAg VLPs were produced in *E. coli*, due to its low production cost, less growth time and simple culture conditions. After purification and self-assembly, the potential of hybrid VLPs as a vaccine against PRRSV was evaluated by testing their ability to block virus infection in MARC 145 cells [17].

## 2. Results

### 2.1. Construction of Hybrid HBcAg VLPs

**Figure 1:**
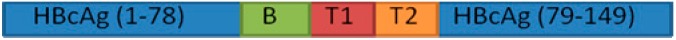
Construction of hybrid HBcAg VLP. B (green color) represents the linear and conserved protective B-cell epitope SHLQLIYNL. T1 represents the conserved protective T-cell epitope KGRLYRWRSPVIIEK. T2 represents the conserved protective T-cell epitope LAALICFVIRLAKNC. There is a His_6_-tag at the *N*-terminal of the protein.

**Figure 2:**
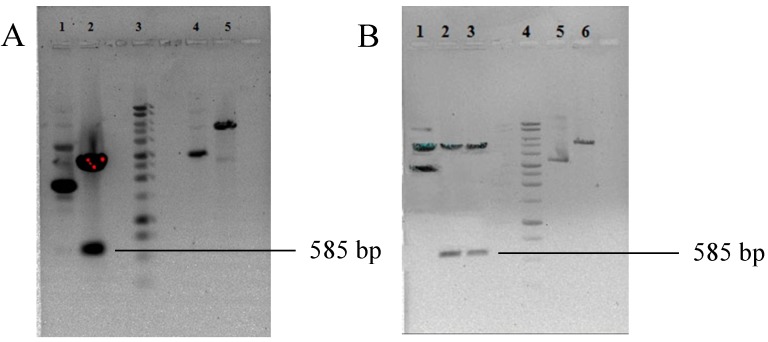
Agarose gel electrophoresis showing the presence of HBcAg VLP in the pUC57 vector and (pET28a/VLP). (**A**) The nucleotide sequence coding for HBcAg VLP from the pUC57 vector. Lane 1: uncut pUC 57 vector containing the HBcAg VLP sequence; Lane 2: pUC57 vector containing the HBcAg VLP sequence digested with NheI and HindIII enzymes; Lane 3: 1-kb DNA ladder; Lane 4: uncut pET28a vector; Lane 5: pET28a vector linearized with NheI and HindIII enzymes; (**B**) Confirmation of the presence of the hybrid HBcAg VLP nucleotide sequence in (pET28a/VLP). Lane 1: uncut (pET28a/VLP); Lanes 2–3: pET28a HBcAg VLP digested with NheI and HindIII enzymes; Lane 4: 1-kb DNA ladder; Lane 5: uncut pET28a vector; Lane 6: pET28a vector with NheI and HindIII enzymes.

The conserved protective epitopes of GP5 were fused at a region located at the tip of the core particle surface spikes of HBcAg, as shown in Figure 1. It has been reported that the region between amino acids 78 and 82 forms the tip of the core particle [18]. A lysine (K) residue was added to the *C*-terminus of the T2 epitope (LAALICFVIRLAKNC) for its efficient processing [19]. The nucleotide sequence coding for this construct was codon optimized using Optimum Gene™ for efficient protein expression in *E. coli*. The codon adaptation index (CAI) was increased from 0.56 to 0.89, where a CAI greater than 0.8 is regarded as good for protein expression in *E. coli*. The codon-optimized nucleotide sequence was artificially synthesized and cloned in the pUC57 vector at the BamHI and HindIII sites. Digestion with NheI and HindIII enzymes released a 585-bp insert. The 585-bp insert release (Lane 2, Figure 2A) and the pET28a vector digested with NheI and HindIII enzymes (Lane 5, Figure 2A) were cloned using T4 DNA ligase enzyme (NEB, Ipswich, MA, USA). The clone was confirmed using double digestion (Figure 2B), where an insert of 585-bp product is seen (Lanes 2 and 3). No insert release is seen in the vector alone in Lane 6, which served as a control.

### 2.2. Expression and Solubility Check of Hybrid HBcAg VLPs

**Figure 3:**
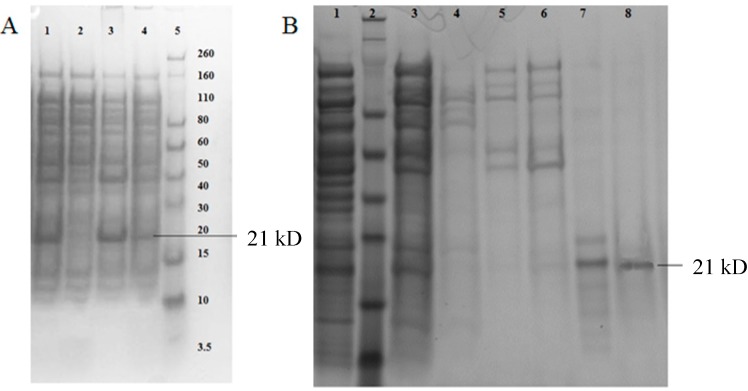
Expression, solubility and purification profile of hybrid HBcAg VLP. (**A**) Expression and solubility profile of hybrid HBcAg VLP protein. Lane 1: total protein fraction; Lane 2: uninduced cell lysate; Lane 3: protein fraction in pellet; Lane 4: soluble protein fraction; Lane 5: pre-stained Novagen marker; (**B**) Purification profile of hybrid HBcAg VLP protein. Lane 1: inclusion body (IB)-solubilized protein fraction; Lane 2: prestained Novex protein marker; Lane 3: ammonium sulfate precipitated protein fraction applied onto immobilized metal affinity chromatography (IMAC) column; Lane 4: unbound protein fraction; Lane 5: 10 mM imidazole wash; Lane 6: 30 mM imidazole wash; Lane 7: elute from IMAC, which is refolded by dialysis; Lane 8: elute fraction from anion exchange (AEX) chromatography.

Kanamycin-resistant clones were examined for hybrid HBcAg VLP protein expression under IPTG induction. Different concentrations of IPTG ranging from 0.3 to 1 mM were used to obtain satisfactory expression of the recombinant protein. *E. coli* cells induced with 1 mM IPTG gave maximum expression with a post-induction temperature of 37 °C for 3.5 h. Figure 3A compares the expression profiles of induced samples with that of un-induced samples and shows a unique protein band at 21 kDa in the total protein of IPTG-induced cells (Lane 1). Unfortunately, almost all recombinant proteins were present in the pellet fraction (Lane 3, Figure 3A). Several methods, such as using different growth media for *E. coli*, lowering the post-induction temperature, using different IPTG concentrations and different buffers with different pH for protein extraction were attempted to make hybrid HBcAg VLP proteins soluble, but none of these methods were successful.

### 2.3. Purification and Refolding of HBcAg VLP Proteins from Inclusion Bodies

After cell growth and lysis, inclusion bodies were solubilized in inclusion body (IB) solubilizing buffer containing 0.9% sarkosyl. As the HBcAg VLP protein has a His-tag at its *N*-terminal, immobilized metal affinity chromatography (IMAC) was used for protein purification. It was found that 30 mM imidazole in the washing buffer was optimal to remove most of the impurities bound to the column (Lane 6, Figure 3B). Elution peak fractions were collected, and the recombinant protein was refolded by stepwise dialysis. Refolding efficiency was found to be 80%. Urea was also used to extract proteins from inclusion bodies. Although purified proteins could be obtained by using 8 M urea, refolding was not successful. The target protein precipitated during stepwise dialysis of urea removal. On column protein refolding using IMAC, Sepharose 6 Fast Flow resin (GE Healthcare, Uppsala, Sweden) was attempted, but also failed. Therefore, using 0.9% sarkosyl in IB solubilizing buffer appears to be the best method for the purification of this particular protein. Refolded HBcAg VLP protein was subjected to anion exchange chromatography to further remove the residual impurities.

**Figure 4:**
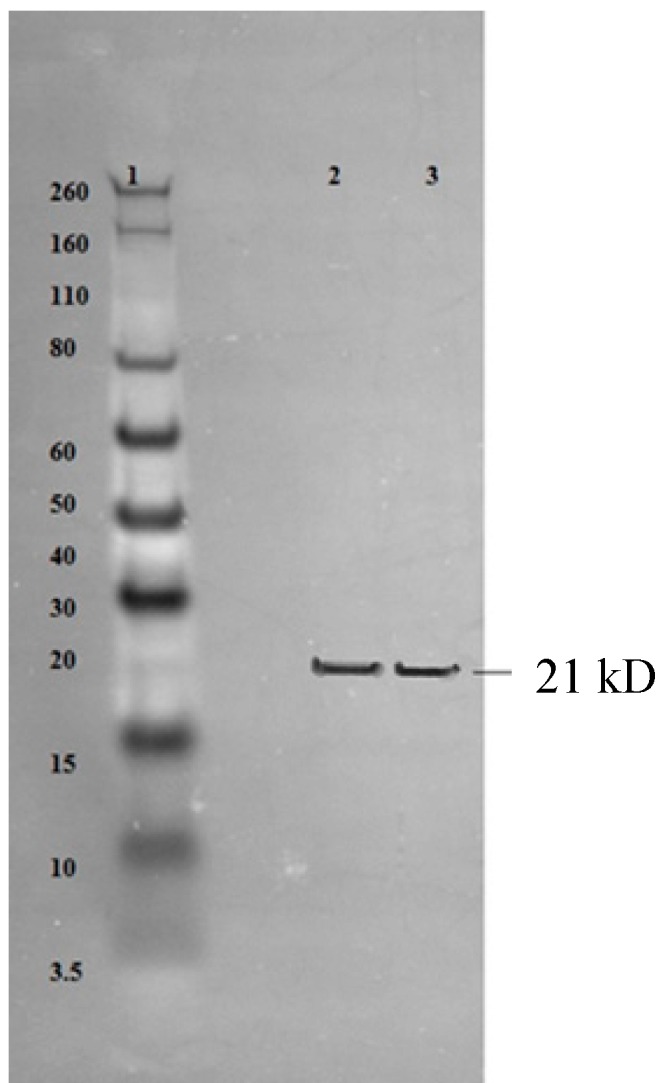
Western blot analysis of hybrid HBcAg VLP protein. Lane 1: prestained Novex protein marker; Lane 2: refolded hybrid HBcAg VLP protein; Lane 3: elute fraction from AEX chromatography.

The elution peak from anion exchange (AEX) chromatography was collected and analyzed on sodium dodecyl sulfate-polyacrylamide gel electrophoresis (SDS-PAGE). A clear single band corresponding to 21 kDa is seen in Lane 8 of Figure 3B. A clear band of a size of 21 kDa is also obtained by Western blot (Figure 4) with anti-His monoclonal antibodies. Based on the results from SDS PAGE and Western blot, it can be concluded that HBcAg VLP protein is of high purity.

**Figure 5:**
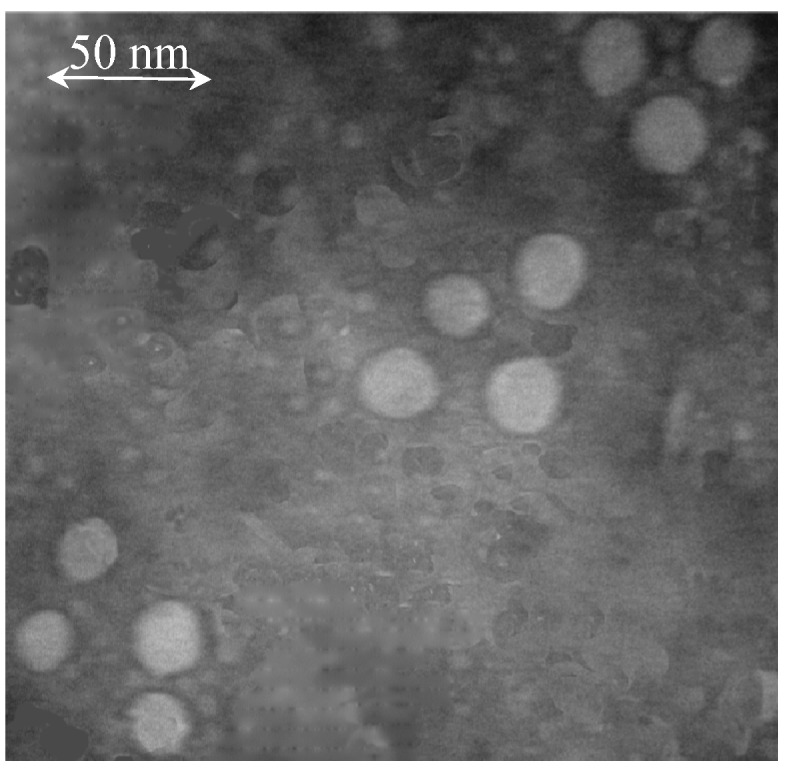
Examination of hybrid HBcAg VLP protein by transmission electron microscopy.

Hybrid HBcAg VLP protein was allowed to stand overnight and examined under transmission electron microscopy. VLPs of a size corresponding roughly to around 23 nm were visualized, as shown in Figure 5, with significant homogeneity. Figure 5 confirms the self-assembly of hybrid HBcAg VLP protein into VLPs. It has been reported earlier that purification of protein with ion exchange chromatography can lead to successful self-assembly of protein into VLPs without the ultracentrifugation process [20].

### 2.4. Virus Blocking Assay and Endotoxin Assay

**Figure 6:**
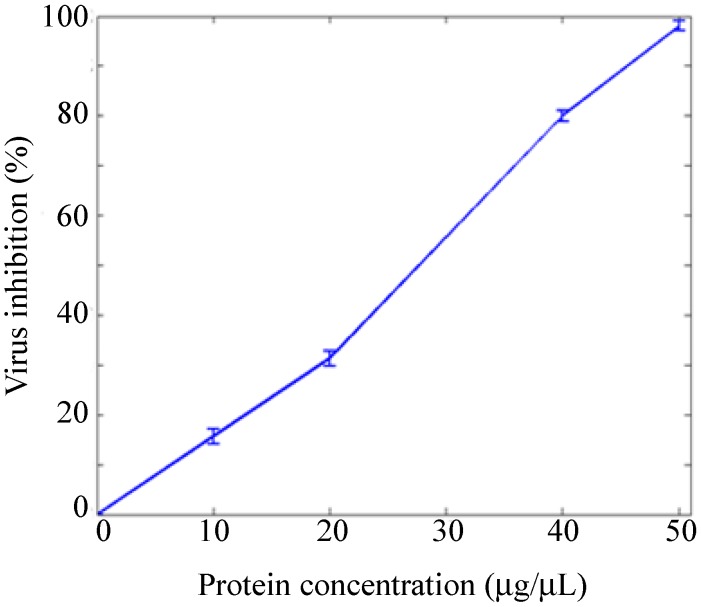
Virus blocking assay of hybrid HBcAg VLPs. The error bar denotes the standard deviation of three replicate counts.

The B-cell epitope and T-cell epitopes are all located in the ectodomains (amino acids 32–60 and 126–200) of GP5, and the ectodomains were shown to contribute to the attachment of the virus to susceptible cells [17]. Thus, a virus blocking assay was conducted to demonstrate that the epitopes are displayed in the principal antigenic tip structure of HBcAg. The virus blocking assay results are shown in Figure 6. Evidently, the virus inhibition rate is positively correlated with the concentration of HBcAg VLP protein, indicating the direct contribution of the protein to the blocked infection of MARC 145 cells by PRRSV. HBcAg VLP protein was examined for endotoxin content, as endotoxins are highly toxic and are known to cause severe reactions in human and animals, even at low concentrations. It was found that the endotoxin level was 5 EU/mg of protein, which is less than the acceptable endotoxin range (6–2000 EU/mg) for drug administration into animals.

## 3. Discussion

PRRSV is highly pathogenic and causes huge economic loses to the pig industry worldwide. Vaccination appears to be the cost-effective and feasible method to control PRRSV. Currently, inactivated vaccines and modified live vaccines (MLV) are commercially available against PRRSV. Inactivated vaccine under the trademark PRRomiSe™, Intervet, was once available in U.S., but has been discontinued since 2005. Nevertheless, inactivated vaccines are ineffective even against homologous strains [8,21]. Several MLV vaccines, such as Ingelvac^®^ PRRS MLV and ReproCyc^®^ PRRS-PLE, are licensed for use in U.S. However, PRRSV MLV vaccines have myriad concerns with respect to efficacy and safety, such as ineffective protection against heterologous strains, reversion of vaccine to pathogenic virus and shedding of infective virus upon vaccination. Several experimental vaccines have been reported, including mixed strain vaccines [22,23], recombinant subunit vaccines bearing different PRRSV structural proteins, such as DNA [24,25], bacteria [26], adenovirus [27,28,29], poxvirus [30,31], alphavirus [32], baculovirus [33] and gastroenteritis virus [34], plant-derived vaccines [35,36,37] and synthetic peptide vaccines [38]. These vaccines can confer protection at its best, but do not possess the characteristics of a universal PRRS vaccine.

VLP-based vaccines are one of the most exciting and emerging vaccine technologies, as they present epitopes in similar patterns as those of native viruses. VLPs are super-molecular assemblages that mimic the structure of authentic viruses, without being infectious. HBcAg VLPs are considered very flexible, as they allow a wide array of foreign insertions [39]. As HBcAg VLPs even elicit strong B-cell and T-cell responses [40], they are regarded as one of the most promising VLP-based vaccine platforms. During the last decade, two prophylactic VLP vaccines for the prevention of hepatitis B virus and human papillomavirus infections have been approved for human use, and another 12 vaccines have entered clinical development [41,42]. A wide variety of VLP-based vaccines have been developed for animals [43]. However, studies on VLP-based PRRSV vaccines are very limited. Recently, VLPs comprised of GP5 and M proteins of PRRSV were generated in insect cells and were shown to induce both neutralizing antibodies and IFN-γ response in mice [16]. However, the existence of adverse factors within viral proteins, such as a non-conserved epitope, which is an immunodominant decoy epitope in viral GP5 protein, and glycan shielding of neutralization epitopes can mislead the immune system to non-conserved epitopes and/or diminish the immune responsiveness of the conserved protective epitopes [44,45]. This may explain the poor heterologous protection of current commercial and experimental PRRS vaccines. Therefore, the key to a universal PRRS vaccine is to establish a method to potentiate the immune response to the conserved protective epitopes of PRRSV and to focus the immune system on these epitopes by effective antigen presentation and removal of the adverse factors within the viral proteins. This study is the first to explore the novel antigen presentation method by fusing the conserved protective epitopes of PRRSV with HBcAg, which will self-assemble to form VLPs.

Encouragingly, although it is partial or weak, heterologous protection does exist, and common epitopes are likely to be involved in protection in different strains [46]. Three conserved protective epitopes (one B-cell epitope and two T-cell epitopes) are used in this study. The B-cell epitope is conserved among isolates, and neutralizing antibodies are mainly directed against this epitope [6,45,47]. The T-cell epitopes (^117^LAALICFVIRLAKNC^131^ and ^149^KGRLYRWRSPVIIEK^163^) are also conserved and can recall IFN-γ response [7]. Epitope-based vaccines are captivating, because of their advantages, such as a high degree of specificity and their ease and safety of use [48]. In this study, hybrid HBcAg VLP proteins were generated by the fusion of three conserved epitopes’ genes into HBcAg at the major immunodominant region (MIR) site, and these hybrid HBcAg VLPs were shown to block virus effectively when tested on MARC 145 cells. As the MIR of HBcAg is the most exposed region of the assembled VLP, those epitopes were more likely to be presented on the surface of each hybrid HBcAg VLP, which might further increase the avidity for the target epitope.

A limit of the current study is the lack of *in vivo* studies to examine the effectiveness of hybrid HBcAg VLPs in mice and in pigs. Although the utility of these epitopes is still debatable [49,50], it has been reported recently that B-cell and T-cell epitopes of GP5 along with adjuvant Gp96N could elicit strong immune responses in mice [51]. It was revealed that Gp96N activated PRRSV-specific humoral responses elicited by B-cell epitope peptides and promoted the PRRSV specific cellular immunity elicited by T-cell epitope peptides [51]. Therefore, hybrid HBcAg VLPs generated in this study should be able to elicit both humoral and cell-mediated immunity in mice and pigs.

## 4. Experimental Section

### 4.1. Construction of Recombinant Plasmids

The conserved protective B-cell and T-cell epitopes were inserted between amino acids 78 and 79 of hepatitis B virus core protein. The nucleotide sequence of 585 bp, coding for this construct, was codon optimized using Optimum Gene™ by Genscript Corporation (Piscataway, NJ, USA). The codon-optimized nucleotide sequence was artificially synthesized and cloned into a pUC57 vector at BamHI and HindIII by Genscript Corporation (Piscataway, NJ, USA). The pUC57 plasmid was digested by NheI and HindIII enzymes, as the NheI restriction site was incorporated at the *N*-terminal of the sequence to assist cloning into a pET28a vector. The constructed plasmid (pET28a/VLP) was transformed into *E. coli* DH5 alpha cells by electroporation. The presence of the 585-bp insert in the constructed plasmid (pET28a/VLP) was confirmed by restriction digestion, and the confirmed plasmid was transformed into *E. coli* BL21 (DE3).

### 4.2. Expression and Solubility Test

Pre-inoculum of *E. coli* transformed with recombinant plasmid (pET28a/VLP) was prepared in 50 mL of 2x YT media supplemented with 50 µg/mL kanamycin. The culture was incubated at 37 °C and 200 rpm for 16 h. The inoculum was prepared in 2-liter flasks using 0.02% of pre-inoculum and was incubated at 37 °C and 200 rpm, until the exponential growth phase was reached (optical density at 600 nm reached 0.6–0.7). The culture was induced with 1 mM IPTG and was incubated for an additional three and half hours at 37 °C and 180 rpm.

Cells were harvested by centrifugation at 6000× *g* for 10 min and were resuspended using lysis buffer (50 mM sodium phosphate, 500 mM sodium chloride, pH 8.2). Cells were lysed by ultrasonication with 30% amplitude at 4 °C for about 55–60 cycles. Each cycle had a pulse of seven seconds succeeded by a rest time of five seconds. Total cell extract was subjected to centrifugation at 12,000× *g* for 10 min to separate soluble and insoluble fractions. All of these fractions were analyzed on SDS gel using non-induced cell lysate as a control.

### 4.3. Solubilization of Inclusion Bodies

The insoluble fraction after cell lysis and centrifugation was washed thrice with washing buffer (50 mM sodium phosphate, 500 mM sodium chloride, 0.1% sarkosyl, pH 8.2). An additional three washes with distilled water were done to obtain pure inclusion bodies (IB). These pure inclusion bodies were resuspended in IB solubilizing buffer (50 mM sodium phosphate, 500 mM sodium chloride, 0.9% sarkosyl, pH 8.2). MgCl_2_ (100 mM), DNase (200 µg/mL), RNase (200 µg/mL) and phenylmethylsulfonyl fluoride (PMSF) (100 µg/mL) were added, and the mixture was incubated at room temperature for three hours with frequent vortexing. Later, the mixture was subjected to centrifugation at 17,000× *g* for 10 min, and the supernatant was collected. The supernatant was precipitated with 30% (NH_4_)_2_SO_4_, and the precipitate was subjected to centrifugation at 17,000× *g* for 10 min. The collected precipitate was dissolved in column loading buffer (50 mM sodium phosphate, 500 mM sodium chloride, 0.9% sarkosyl, pH 8.2).

### 4.4. Immobilized Metal Ion Chromatography

An ÄKTA™ purifier fast performance liquid chromatography (FPLC) system (GE Healthcare, Uppsala, Sweden) was used for all protein purification experiments. A column with 5 mL of immobilized metal ion chromatography (IMAC) Sepharose 6 Fast Flow resin (GE Healthcare) was prepared as per the manufacturer’s instructions. The resins were charged with 0.1 M NiSO_4_ and equilibrated with 10 column volumes (CV) of column loading buffer. Five milliliters of the precipitate dissolved in column loading buffer were loaded onto the column. Unbound proteins were removed by 10 CV of loading buffer, and finally, the bound proteins were eluted by elution buffer (50 mM sodium phosphate, 500 mM sodium chloride, 0.9% sarkosyl, 300 mM imidazole, pH 8.2).

### 4.5. Refolding of Recombinant Fusion Proteins

IMAC elutes were pooled and dialyzed at room temperature against refolding buffer using Slide-A-Lyzer Dialysis Cassettes with a molecular weight cut-off of 3500 (Thermo Scientific, Rockford, IL, USA). The refolding buffers were 0.5%, 0.25%, 0.05% sarkosyl with 25 mM sodium phosphate and 100 mM sodium chloride (pH 8.2). Protein samples were dialyzed with each concentration of sarkosyl for 8 h.

### 4.6. Anion Exchange Chromatography

Final polishing of refolded recombinant fusion protein was performed using anion exchange chromatography (AEX). A column with 5 mL of DEAE Sepharose Fast Flow resin (GE Healthcare) was packed as per the manufacturer’s instructions. The column was equilibrated with 10 CV of AEX binding buffer (25 mM sodium phosphate, 100 mM sodium chloride, 0.05% sarkosyl, pH 8.2), and 5 mL of refolded protein sample was loaded onto the column. Unbound proteins were removed by washing with 5 CV of AEX binding buffer. Elution of bound protein was performed using a NaCl gradient starting with 100 to 500 mM along with 25 mM sodium phosphate and 0.05% sarkosyl (pH 8.2). Finally, the column was regenerated using 1 M NaCl and 25 mM sodium phosphate, pH 8.2.

### 4.7. SDS PAGE and Western Blotting

Briefly, lysates samples were separated by 12% NuPAGE Bis-Tris gel and were transferred on to a nitrocellulose membrane (Whatman, Dassel, Germany) as per the manufacturer’s instruction. The membrane was blocked with 5% nonfat dry milk in Tris-buffered saline (TBS) for 1 h at room temperature. It was then incubated with a primary antibody, anti-His monoclonal antibody, THE™ Anti-His mAb, Mouse (subtype IgG1) (Genscript Corporation) 1:2000 dilution, in TBS containing 5% nonfat dry milk for 1 h at room temperature. The membrane was then washed thrice with TBS buffer with 0.02% of Tween 20 (TBST) for 10 min each. Later, the membrane was incubated with a secondary antibody, horse radish peroxidase (HRP) goat anti-mouse IgG antibody (Bethyl Laboratories Inc., Montgomery, TX, USA) 1:20,000 dilution, in TBS containing 5% nonfat dry milk for 1 h at room temperature. The membrane was then washed thrice with TBST for 10 min each. Later, the membrane was incubated in a 1:1 mixture of HRP luminal/enhancer solution and peroxide buffer (Bio-Rad, Hercules, CA, USA) for 5 min. The membrane was visualized by a ChemiDoc XRS molecular imager (Bio-Rad).

### 4.8. Electron Microscopy

Purified VLPs (2–3 µg) were adsorbed on a freshly carbon-coated grid. Excess sample was blotted by filter paper and allowed to stand for 1 min. Later, the sample was stained with freshly prepared 1% aqueous uranyl acetate. Excess stain was blotted using filter paper, and samples were allowed to dry. For transmission electron microscopy (TEM), a JEM 1400 manufactured by JEOL (Tokyo, Japan) was used. Micrographs were taken under 20,000× magnification.

### 4.9. Virus Blocking Assay

To analyze the ability of purified hybrid HBcAg VLPs to block PRRSV-susceptible cells, an indirect immune-fluorescence test was performed as described elsewhere with some modifications [17]. Purified hybrid HBcAg VLPs were added into wells of a plate coated with confluent MARC-145 cells. After incubation for 1 h, PRRSV (VR2385), dilutions with 2000 TCID_50_/mL were added to each well and incubated for 15 min at 37 °C. The inoculums were then removed, and 100 μL of fresh Dulbecco’s Modified Eagle Medium (DMEM) supplemented with 2% fetal bovine serum were added to each well. After incubation for 24 h at 37 °C in a humidified atmosphere of 5% CO_2_, the plates were fixed with 80% acetone for 15 min at room temperature. After extensive washing with phosphate buffered saline with 0.05% of Tween 20 (PBST), 50 μL of fluorescein isothiocyanate-conjugated anti-PRRSV monoclonal antibody, SDOW17-F (Rural Technologies, Brookings, SD, USA), diluted 1:100 in PBST with 2% BSA was added to each well. After an hour of incubation, the cells were washed four times with PBS, and the number of fluorescent foci in each well was counted. The virus inhibition rate was expressed as the ratio of reduced fluorescent foci number by a protein sample over the number of fluorescent foci of the negative control (all assay components without hybrid HBcAg VLPs).

### 4.10. Endotoxin Assay

The endotoxin level of purified refolded protein was determined using the ToxinSensor™ chromogenic limulus amebocyte lysate (LAL) endotoxin assay kit, according to manufacturer’s instructions (Genscript Corporation, Piscataway, NJ, USA).
